# Integrative Analysis of Immune- and Metabolism-Related Genes Identifies Robust Prognostic Signature and PYCR1 as a Carcinogenic Regulator in Clear Cell Renal Cell Carcinoma

**DOI:** 10.3390/ijms26104953

**Published:** 2025-05-21

**Authors:** Guo Zhao, Jiatong Ding, Jiaxiu Ma, Yale Jiang, Yuning Wang, Shuhang Wang, Ning Li

**Affiliations:** 1 Clinical Trial Center, National Cancer Center/National Clinical Research Center for Cancer/Cancer Hospital, Chinese Academy of Medical Sciences and Peking Union Medical College, Beijing 100021, China; s2023003010@pumc.edu.cn (G.Z.); dingjiatong9@foxmail.com (J.D.); yale_jiang@163.com (Y.J.); yuningwang1998@163.com (Y.W.); 2 State Key Laboratory of Experimental Hematology, National Clinical Research Center for Blood Diseases, Haihe Laboratory of Cell Ecosystem, Institute of Hematology and Blood Diseases Hospital, Chinese Academy of Medical Sciences and Peking Union Medical College, Tianjin 300052, China; majiaxiu@ihcams.ac.cn

**Keywords:** clear cell renal cell carcinoma, immune cell, metabolism, *PYCR1*

## Abstract

Clear cell renal cell carcinoma (ccRCC) is distinguished by metabolic irregularities and unique immunological profiles. Nevertheless, the comprehensive examination of immune and metabolic attributes within the tumor microenvironment of ccRCC remains inadequately elucidated. In this study, we identified two distinct molecular subtypes (C1 and C2) of ccRCC using the non-negative matrix factorization (NMF) algorithm. Utilizing univariate and least absolute shrinkage and selection operator (LASSO) Cox regression analyses, we developed a prognostic signature comprising eight immune- and metabolism-related genes (IMRGs) associated with the tumor microenvironment. The validation of this signature was performed using both testing and entire datasets. A nomogram was developed using IMRGs prognostic signature and various clinical parameters, including age and TNM stage. We also performed the in vitro experiments to validate the carcinogenic role of PYCR1 in ccRCC cells. Subtype C1 exhibited a more favorable prognosis and higher levels of immune cell infiltration compared to subtype C2. The AUCs of the nomogram at 1-, 3-, and 5-year intervals (AUC = 0.874, 0.820, and 0.794) were slightly higher than those of the IMRGs signature alone (AUC = 0.773, 0.755, and 0.764). The association between risk score and immune checkpoint expressions, immunophenoscore (IPS), and microsatellite instability (MSI) collectively predicted treatment efficacy accurately. Additionally, in vitro experiments confirmed the involvement of *PYCR1* in promoting the aggressive behaviors of ccRCC cells, as evidenced by reduced proliferation, invasion, and enhanced apoptosis upon *PYCR1* knockdown. In conclusion, the IMRGs signature shows promise in predicting prognostic risk, assessing the effectiveness of immunotherapy, and tailoring treatment for ccRCC patients.

## 1. Introduction

Tumor cells develop sophisticated strategies to circumvent immune surveillance, primarily through the secretion of immunosuppressive mediators and recruitment of regulatory immune cells, collectively fostering a supportive tumor microenvironment (TME) that facilitates malignant progression [1]. Concurrently, metabolic reprogramming emerges as a fundamental adaptive mechanism in oncogenesis, empowering neoplastic cells to thrive under hypoxic, acidic, and nutrient-deficient conditions [2]. This metabolic plasticity extends beyond cellular autonomy to profoundly influence T cell immunobiology, modulating effector responses, exhaustion states, cellular senescence, and memory differentiation patterns—all critical determinants of anti-tumor immunity [3,4]. The dynamic crosstalk between tumor-intrinsic metabolic adaptations and systemic immune regulation carries substantial translational implications for cancer management, particularly in therapeutic stratification [5]. Clinically, colorectal cancers demonstrating elevated metabolic–immune prognostic signatures correlate with heightened tumor mutational burden and robust immunosuppressive networks, factors potentially dictating heterogeneous responses to immune checkpoint blockade therapies [6]. This intricate crosstalk underscores the necessity for dual-targeting approaches that concurrently address metabolic plasticity and immune dysfunction in precision oncology [7].

As the predominant subtype accounting for 75% of renal tumors, clear cell renal cell carcinoma (ccRCC) develops an immunologically active yet functionally impaired TME, where abundant infiltrating immune cells paradoxically exhibit suppressed effector functions that ultimately enable tumor immune evasion [8]. This immunological paradox is amplified by the marked interpatient heterogeneity in clinical responses to immune checkpoint inhibitors (ICIs) observed in advanced ccRCC, necessitating molecular stratification strategies to identify immunotherapy-responsive subgroups through systematic characterization of immune evasion-associated genetic signatures [9]. The biological complexity extends to hallmark metabolic reprogramming events in ccRCC, which not only sustain tumor proliferation but also orchestrate immunosuppressive networks and modulate therapeutic sensitivity to targeted agents [10]. Multi-omic integration has elucidated the multidimensional crosstalk between metabolic and immune landscapes, where heightened pyrimidine/cytidine metabolic flux correlates with CD8^+^ T cell infiltration patterns and unfavorable clinical outcomes [11]. Other multi-omic profiling of ccRCC also identified that there are distinct subtypes with distinctive metabolic features and immune microenvironment in ccRCC, highlighting the intra-tumoral heterogeneity [12,13,14]. The convergence of immune escape mechanisms and metabolic plasticity fundamentally dictates disease progression trajectories and therapeutic vulnerabilities in ccRCC. Advancing precision medicine in this malignancy requires the mechanistic dissection of these intertwined pathways to develop predictive biomarkers and rationally designed combination therapies targeting both immunological and metabolic axes of tumor adaptation.

Pyrroline-5-carboxylate reductase (PYCR) family enzymes, particularly the isoform PYCR1 that drives the terminal step of proline biosynthesis, exhibit frequent oncogenic overexpression across malignancies and demonstrate strong associations with adverse clinical trajectories, positioning them as promising diagnostic biomarkers and intervention targets [15,16]. In ccRCC pathogenesis, PYCR1 exerts dual oncogenic functions by fueling tumor proliferation and metastatic dissemination through mTOR signaling activation while concurrently shaping the immune landscape via its correlation with the enhanced infiltration of activated CD4^+^ memory T cells—a paradoxical relationship suggesting metabolic–immune crosstalk within the tumor microenvironment [17]. This dual role in coordinating tumor metabolism and immunomodulation has propelled PYCR1 into the forefront of translational oncology research, though mechanistic details of its tumorigenic circuitry and therapeutic vulnerability remain to be fully deciphered [18,19].

To further link the immune- and metabolic features of ccRCC, we implemented a multi-dimensional bioinformatics analysis and experimental validation leveraging TCGA KIRC datasets. Through molecular subtyping based on immune–metabolic gene signatures, we stratified patients into distinct prognostic clusters and established an eight-gene immune–metabolism-related prognostic signature (IMRG) with validated predictive capacity for clinical outcomes. Subsequent characterization revealed intricate connections between IMRG profiles, immune microenvironment features, and immunotherapy responses patterns. Focusing on PYCR1 as a prioritized IMRG candidate due to its high expression in ccRCC cell lines, we conducted preclinical ccRCC cells to unravel its pathobiological contributions. Our integrative approaches not only advance the molecular taxonomy of ccRCC but also provide a prototype for developing prognostic biomarkers and metabolism–immunity co-targeting strategies, potentially informing personalized therapeutic decisions in this challenging malignancy.

## 2. Results

### 2.1. Identification of ccRCC Subtypes with Prognosis Differences Based on Immune- and Metabolism-Related Genes

**Figure 1:**
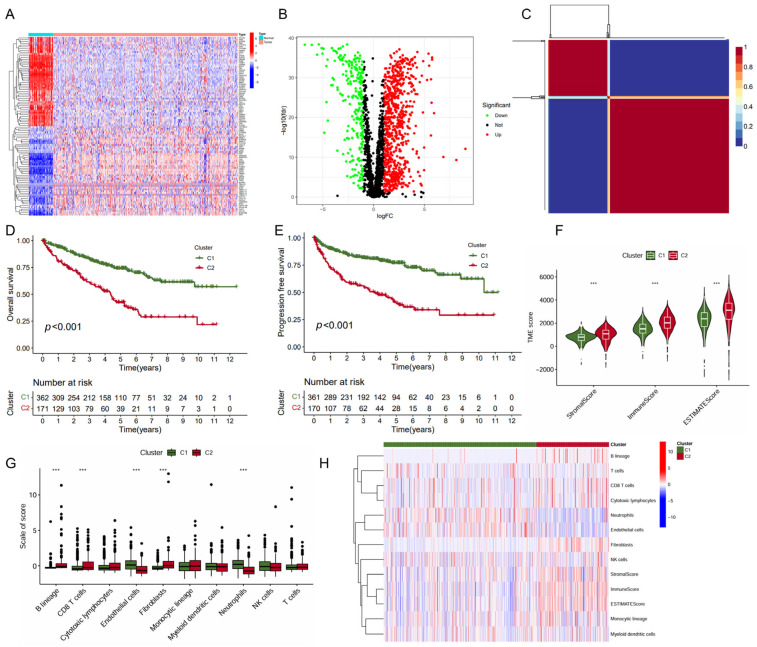
Identification of KIRC molecular subtypes based on NMF algorithm. (**A**) Heatmap of immune- and metabolism-related gene expression profiling between the tumor and normal tissues; (**B**) volcano plot of differential gene expression between the tumor and normal tissues; (**C**) heatmap of nsNMF consensus matrix of K = 2; and (**D**,**E**) Kaplan–Meier curve of OS (**D**) and PFS (**E**) for KIRC subtypes. (**F**) Comparison of immune scores between the two subtypes using “ESTIMATE” algorithm. (**G**) Comparison of immune scores calculated by “MCP counter” algorithm between the two subtypes. (**H**) Heatmap of the immune scores for ESTIMATE and MCP counter algorithms. *** *p* < 0.001.

In this study, we collected genes related to immune metabolism and compared the expression differences of these genes between tumor tissues and normal tissues in ccRCC. The heatmap displayed the expression levels of these genes across various samples (Figure 1A). The volcano plot showcased 560 DEGs identified with 440 upregulated DEGs and 120 downregulated DEGs (Figure 1B). Further analysis using the non-negative matrix factorization algorithm stratified ccRCC into two distinct molecular subtypes (C1 and C2) (Figure 1C). Notably, the C1 subtype was associated with significantly improved OS (*p* < 0.001, Figure 1D) and PFS (*p* < 0.001, Figure 1E). The subsequent analysis of the TME scores revealed that subtype C1 exhibited a significantly lower score in StromalScore, ImmuneScore, and ESTIMATEScore compared to subtype C2 (Figure 1F), suggesting a differential composition of the TME between the two subtypes. In line with this, the C1 subtype exhibited significantly higher level of endothelial cells and neutrophils infiltration, but a lower level of B lineage, CD8^+^ T cells, and fibroblasts (Figure 1G,H). Together, these data indicated that subtype C1 showed a more favorable prognosis and higher levels of immune cell infiltration than subtype C2.

### 2.2. Construction of a Prognostic Signature with Immune- and Metabolism-Related Genes

**Figure 2:**
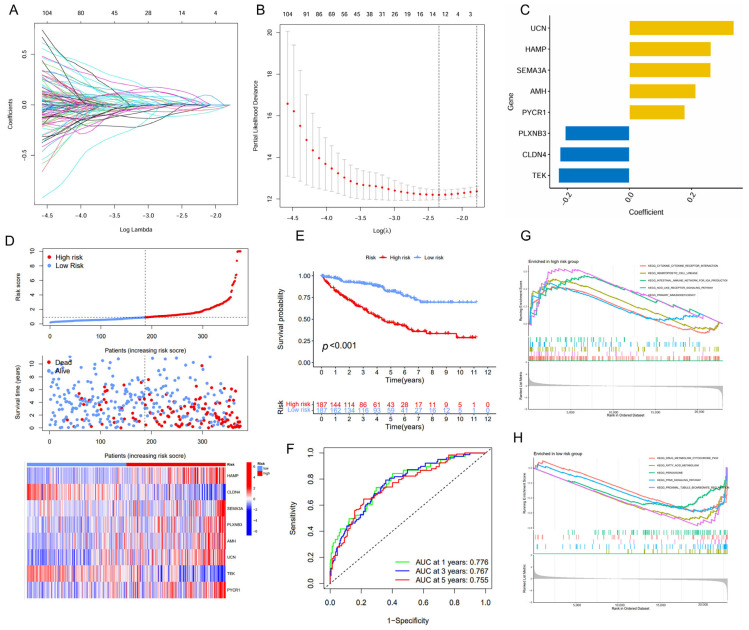
Establishment of the IMRGs prognostic signature using LASSO Cox regression analysis in TCGA KIRC training cohort. (**A**) Coefficients of independent variables in LASSO regression; (**B**) the optimal log value of lambda was indicated by the first black dotted line from the left; (**C**) detailed gene name in the risk model and the coefficient values; (**D**) association between risk scores and patient survival: the top panel displays risk score levels range from low to high; the middle panel presents the distribution of survival time and survival status in relation to risk score across different samples; and the bottom panel features a heatmap, representing the expression levels of the risk model; (**E**) Kaplan–Meier curve analyses for the high-risk group and low-risk group were classified according to the median risk score; (**F**) the ROC curves of the IMRGs prognostic signature at 1-, 3-, and 5-year intervals; and (**G**,**H**) GSEA enrichment analysis in the high-risk group (**G**) and low-risk group (**H**).

The univariate and LASSO Cox regression analyses were employed to identify a subset of prognostic genes associated with ccRCC from a panel of the DEGs (Figure 2A,B). Finally, we conducted a prognostic signature comprising eight IMRGs, including *UCN*, *HAMP*, *SEMA3A*, *AMH*, *PYCR1*, *PLXNB3*, *CLDN4*, and *TEK*, highlighting a strong association between immune metabolism and ccRCC pathogenesis (Figure 2C, Table 1). There is a noticeable trend where high-risk patients have shorter survival times compared to low-risk patients. Additionally, each gene within the IMRGs exhibited distinct expression patterns across high and low-risk patient groups, further confirming their potential associations with prognosis (Figure 2D). The Kaplan–Meier curves also indicated that patients with a low risk exhibited a higher survival probability relative to patients with a high risk, suggesting a strong correlation between the risk score and survival outcomes (Figure 2E). The predictive accuracy of the IMRGs-based model was assessed using time-dependent ROC curves, which yielded area under the curve (AUC) values of 0.776, 0.767, and 0.755 for 1-, 3-, and 5-year overall survival, respectively (Figure 2F). In the testing and entire cohorts, IMRGs demonstrated similar prognostic correlation and predictive power for survival outcomes for ccRCC (Figures S1 and S2). The GSEA analysis provided further insights, indicating that the high-risk group was enriched in pathways such as cytokine–cytokine receptor interaction, hematopoietic cell lineage, intestinal immune network for IgA production, NOD-like receptor signaling pathway, and primary immunodeficiency (Figure 2G), while the low-risk group was enriched in drug metabolism cytochrome P450, fatty acid metabolism, peroxisome, PPAR signaling pathway, and proximal tubule bicarbonate reclamation (Figure 2H).

### 2.3. IMRG Score Correlates with Patient Clinicopathologic Characters

**Figure 3:**
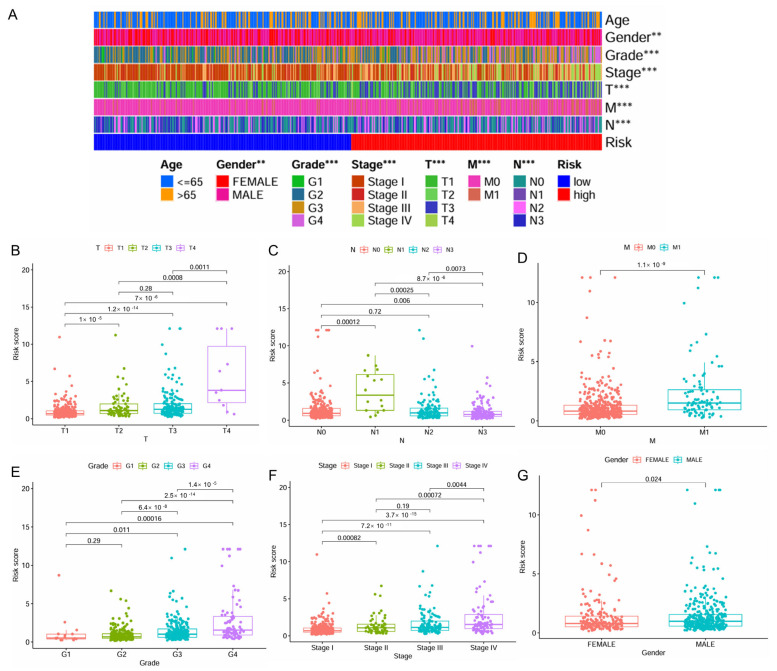
Correlation of the IMRGs prognostic signature with clinical features in the TCGA KIRC cohort. (**A**) The difference in risk scores between different clinical features; (**B**) the difference in risk scores between different T stages; (**C**) the difference in risk scores between different N stages; (**D**) the difference in risk scores between different M stages; (**E**) the difference in risk scores between different tumor grades; (**F**) the difference in risk scores between different tumor stages; and (**G**) the difference in risk scores between patient’s gender. ** *p* < 0.01 and *** *p* < 0.001.

The distribution of patient clinicopathologic characters across the high- and low-risk patient groups was presented in Figure 3A. A significant correlation was observed between higher levels of risk scores and advanced stages of ccRCC, including higher T stage, N stage, M stage, grade, and pathologic stage, as well as male gender (Figure 3B–G). Interestingly, for N stage classification, the risk score reached its peak at the N1 stage, followed by a noticeable decrease (Figure 3C).

### 2.4. IMRG Score Correlates with Immune Microenvironments and Responses to Immunotherapy

**Figure 4:**
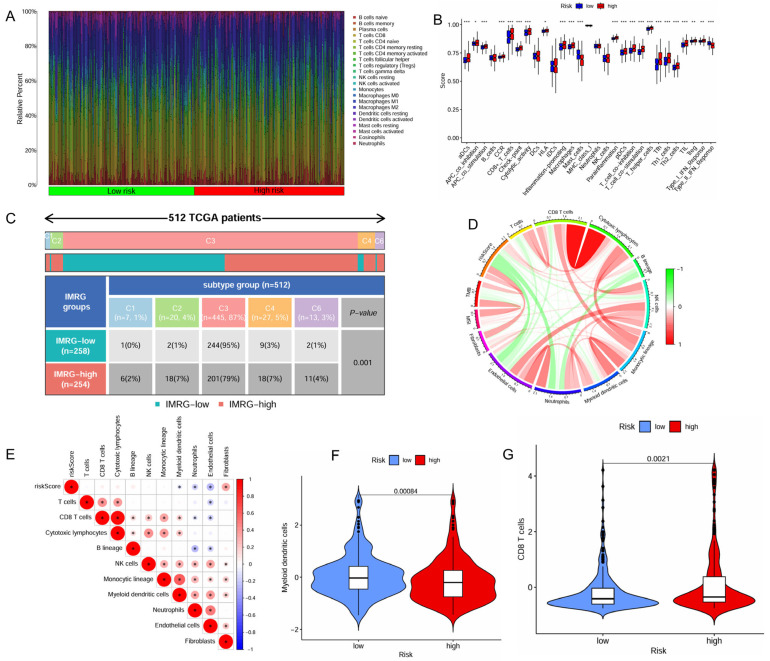
Correlation of the IMRGs prognostic signature with immune microenvironment in the TCGA KIRC cohort. (**A**) Related percent of various immune cells in high- and low-risk groups based on CIBERSORT algorithms; (**B**) differences in immune cells between high- and low-risk groups; (**C**) relationship of immune subtypes [wound healing (C1), IFN-γ dominant (C2), inflammatory (C3), lymphocyte depletion (C4), and TGF-β dominant (C6)] and IMRGs prognostic signature; (**D**) correlation of the risk score, microsatellite instability, tumor mutational burden, and various immune cells; (**E**) correlation analysis of various immune cells and risk scores; (**F**) differences in myeloid dendritic cells between high- and low-risk groups; and (**G**) differences in CD8T cells between high- and low-risk groups. * *p* < 0.05, ** *p* < 0.01, and *** *p* < 0.001.

The distinct immune cell infiltration patterns between the high- and low-risk patient groups is presented in Figure 4A. The high-risk patient group appeared to exhibit a more active immune response, with significantly higher scores across multiple immune functions and cell types, including aDCs, APC co-inhibition, APC co-stimulation, CCR, CD8^+^ T cells, check-point, cytolytic activity, HLA, inflammation-promoting, macrophages, parainflammation, pDCs, T Cell co-inhibition, T Cell co-stimulation, T helper cells, Tfh, Th1 cells, Th2 cells, TIL (tumor-infiltrating lymphocytes), Treg (regulatory T cells), and Type I IFN response (Figure 4B). Furthermore, the high-risk patient group exhibited significantly lower infiltration levels of myeloid dendritic cells but higher infiltration levels of CD8^+^ T cells (Figure 4F,G).

**Figure 5:**
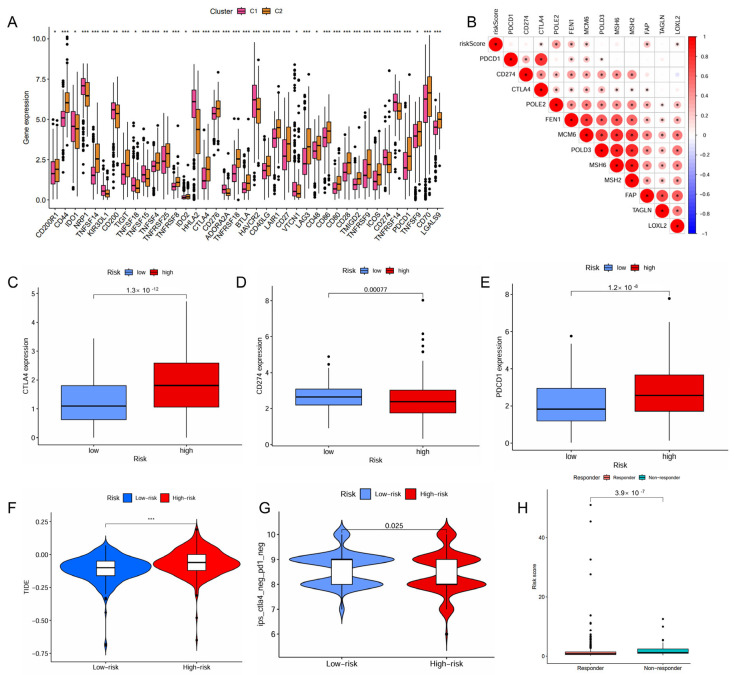
Predictive role of the IMRGs prognostic signature in response to immunotherapy. (**A**) correlation of the different molecule subtypes and immune check point expressions; (**B**) correlation of the risk scores and immune check point expressions; (**C**–**E**) comparison of the difference in immune checkpoint expressions CTLA4 (**C**), CD274 (**D**), and PDCD1 (**E**), between high- and low-risk groups. (**F**) TIDE score of high-risk and low-risk groups; (**G**) correlation between the risk scores and IPS score related to a single immune checkpoint inhibitor (ICI) or a combination of ICIs including PD-1 and CTLA-4; and (**H**) the responses to ICIs of high-risk and low-risk groups. * *p* < 0.05, ** *p* < 0.01, and *** *p* < 0.001.

The high-risk patient group also had a higher proportion than the low-risk group in the C4 and C6 immune subtypes that were associated with a poor prognosis (Figure 4C). The risk score positively correlated with the infiltration levels of fibroblasts, tumor mutational burden (TMB), and microsatellite instability (MSI), but negatively associated with the infiltration levels of myeloid dendritic cells, endothelial cells and neutrophils (Figure 4D,E). The high-risk patient group exhibited a significantly higher TMB (*p* = 0.0061, Figure S3A), indicating a greater potential for immune recognition and response. The risk score was positively correlated with TMB (R = 0.2, *p* = 0.00016, Figure S3B). High TMB (*p* < 0.001) and high TMB combined with high risk (*p* < 0.001) were negatively associated with survival. Patients with high risk and high TMB had the worst prognosis (Figure S3C,D). The expression of immune checkpoints also showed significant differences between the two groups with the risk score demonstrating a positive correlation with CTLA4, POLE2, FEN1, MCM6, FAP, and LOXL2 (Figure 5A,B). The high-risk patient group exhibited higher expression levels of CTLA4 and PDCD1, along with an increased TIDE score and CTLA4-PD1-based IPS, but a lower expression of CD274 (Figure 5C–G). Furthermore, the non-responders of ICIs to immunotherapy tended to present with a higher risk score, suggesting a potential link between risk score and immunotherapy sensitivity (Figure 5H).

### 2.5. Construction and Evaluation of the IMRG-Based Nomogram

**Figure 6:**
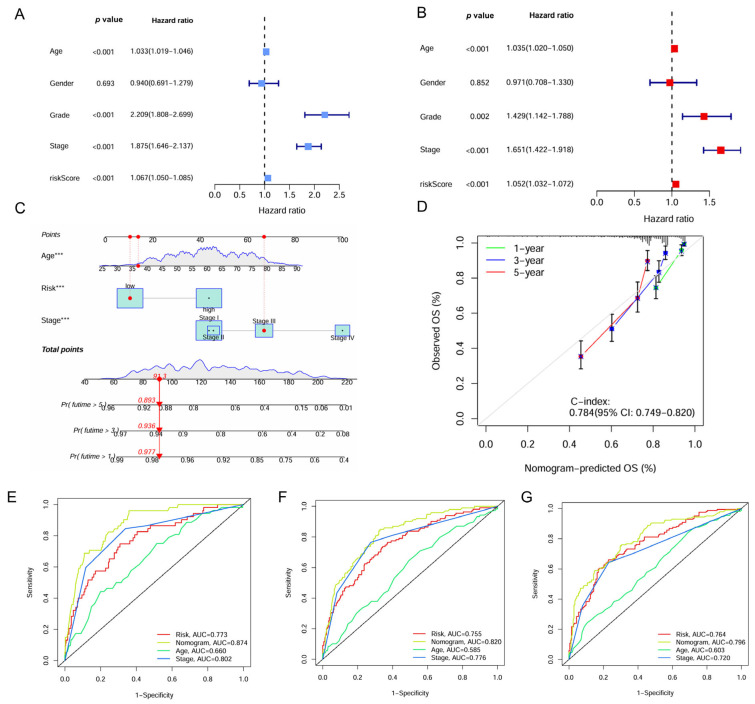
Construction of the nomogram based on the IMRGs prognostic signature and evaluation of clinical significance in the TCGA KIRC cohort. (**A**,**B**) Univariate and multivariate Cox regression analyses of risk score and various clinical features. (**C**) Nomogram for predicting the OS in the entire TCGA KIRC cohort at 1, 3, and 5 years. For each patient, the total score was calculated by adding the points determined by the point scale of each variable. Based on the total points, the bottom scale was used to predict the probability of 1-, 3-, or 5-year survival. The red line exemplified the calculation process and principle of the nomogram. (**D**) Calibration curve for consistency between 1-, 3-, or 5-year nomogram predicted survival and actual survival. (**E**–**G**) ROC curves of nomograms, risk score and other clinical features for 1-year, 3-year, and 5-year survival. *** *p* < 0.001.

Univariate and multivariate Cox regression analyses identified the IMRG signature as an independent prognostic factor for ccRCC with hazard ratios (HRs) of 1.067 (95% confidence interval [CI], 1.050–1.085, *p* < 0.001) and 1.052 (95% CI, 1.032–1.072, *p* < 0.001), respectively (Figure 6A,B). A nomogram was constructed for predicting survival outcomes based on age, risk score, and stage (Figure 6C). The calibration curve demonstrated good concordance between observed and predicted overall survival rates at 1, 3, and 5 years with a C-index of 0.784 (Figure 6D). The ROC curve showed the superior predictive accuracy of nomogram over individual factors including age, risk score, and stage for 1-, 3-, and 5-year survival, with AUC values of 0.874, 0.820, and 0.796, respectively (Figure 6E–G).

### 2.6. Correlation Between IMRG and Drug Sensitivity

**Figure 7:**
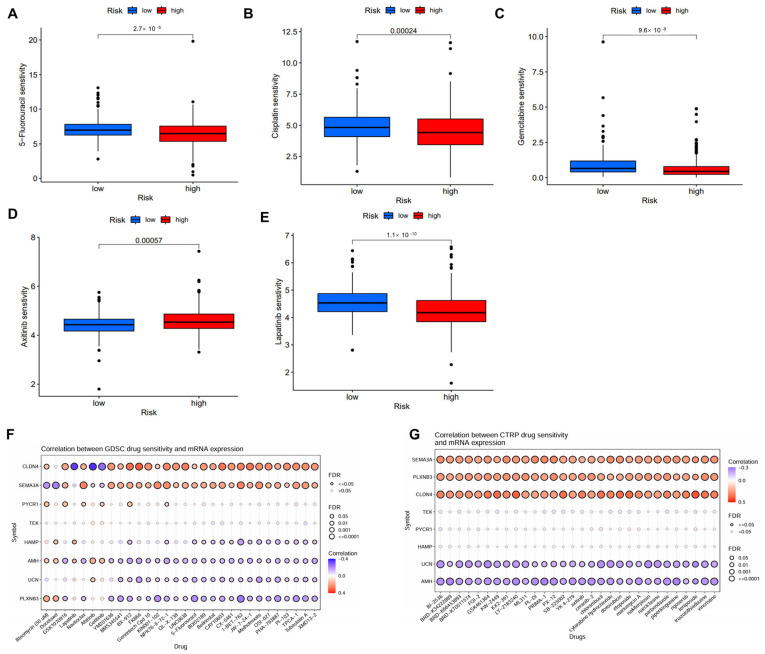
Correlation of the IMRGs prognostic signature and drug sensitivity. (**A**–**E**) Comparison of various drug sensitivities, including 5-fluorouracil (**A**), cisplatin (**B**), gemcitabine (**C**), axitinib (**D**), and lapatinib (**E**) in high- and low-risk groups; (**F**) correlation between GDSC drug sensitivity and mRNA expression of risk model; and (**G**) correlation between CTRP drug sensitivity and mRNA expression of risk model.

Several potential small molecular inhibitors were identified using data from GDSC and CTRP datasets [20,21]. The low-risk patient group exhibited enhanced sensitivity to chemotherapeutic agents such as 5-fluorouracil, cisplatin, gemcitabine, lapatinib, but reduced sensitivity to axitinib (Figure 7A–E). The further analysis of drug sensitivity indicated that the mRNA expression levels of *CLDN4* and *SEMA3A* were positively correlated with the majority chemotherapy drugs in the GDSC database, whereas *AMH*, *UCN*, and *PLXNB3* mainly exhibited negative correlations (Figure 7F). In the CTRP database, *SEMA3A*, *PLXNB3*, and *CLDN4* demonstrated positive correlations with all chemotherapy drugs, while UCN and AMH exhibited negative correlations (Figure 7G).

### 2.7. Knockdown of PYCR1 Inhibits the Malignant Behaviors in ccRCC

**Figure 8:**
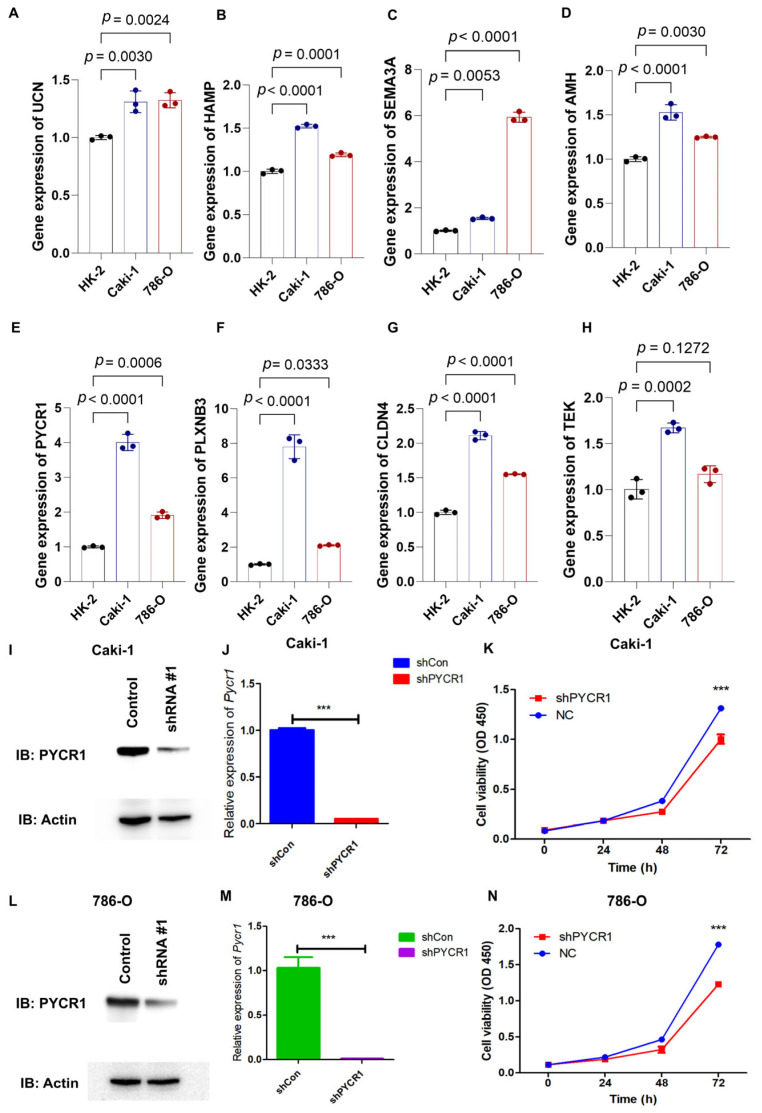
qRT-PCR analysis of genes from risk model, validation of shPYCR1, and cell proliferation essays. (**A**–**H**) Gene expression of *UCN*, *HAMP*, *SEMA3A*, *AMH*, *PYCR1*, *PLXNB3*, *CLDN4*, and *TEK* was detected by qRT-PCR in HK-2, Caki-1, and 786-O cells; (**I**,**J**) fluorescence quantitative PCR detection of shPYCR1 Caki-1 cells; (**K**) Caki-1 cells transfected with PYCR1 siRNAs were subjected to the CCK-8 assay after transfection; (**L**,**M**) fluorescence quantitative PCR detection of shPYCR1 786-O cells; and (**N**) Caki-1 cells transfected with PYCR1 siRNAs were subjected to the CCK-8 assay after transfection. *** *p* < 0.001.

Through qRT-PCR analysis, the mRNA expression profiles of *AMH*, *CLDN4*, *HAMP*, *SEMA3A*, *PLXNB3*, *UCN*, *PYCR1*, and *TEK* showed marked upregulation in 786-O and Caki-1 renal carcinoma cell lines relative to the HK-2 normal renal tubular cell line (Figure 8A–H). PYCR1 emerged as a prioritized candidate for functional characterization in ccRCC pathogenesis based on its pronounced overexpression across both malignant cell models. Targeted shRNA-mediated knockdown successfully attenuated PYCR1 expression at both transcriptional and translational levels in Caki-1 (Figure 8I,J) and 786-O (Figure 8L,M) cells, with validation data presented in Supplementary Figure S4. Functional interrogation through CCK-8 assays revealed the substantial impairment of cellular viability following PYCR1 suppression in both cell lines (Figure 8K,N).

**Figure 9:**
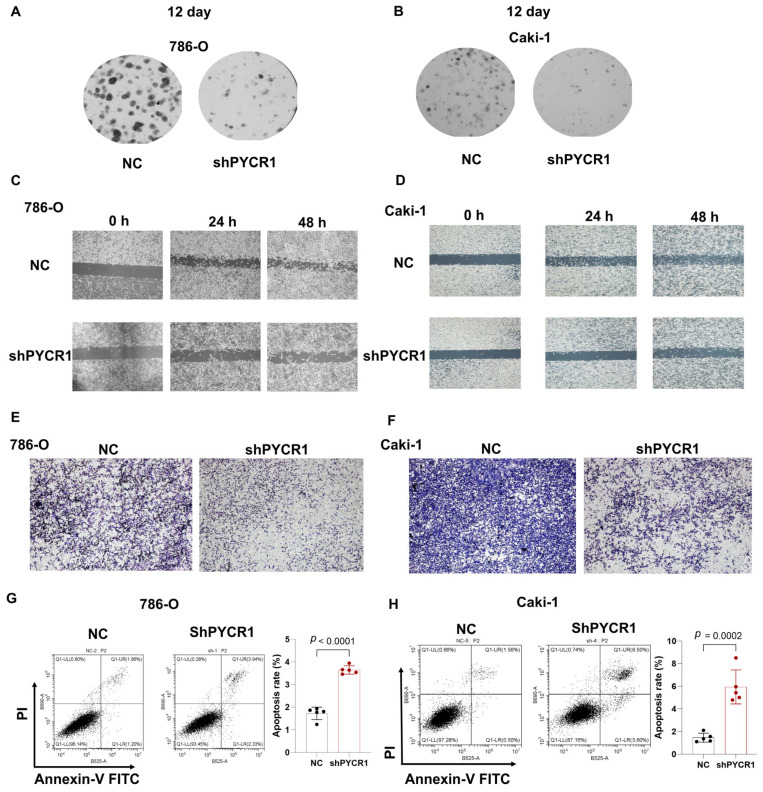
Knockdown of *PYCR1* inhibited colony formation, cell migration, invasion and enhanced cell apoptosis of KIRC cells. (**A**,**B**) 786-O (**A**) and Caki-1 (**B**) cells transfected with PYCR1 siRNAs were seeded onto 6-well plates. The number of colonies was counted on the 12th day after seeding; (**C**,**D**) wound healing ability of 786-O cells (**C**) and Caki-1 (**D**) cells transfected with PYCR1 siRNAs or none after 0, 24, and 48 h; (**E**,**F**) Transwell analysis of 786-O cells (**E**) and Caki-1 (**F**) cells transfected with PYCR1 siRNAs or none; (**G**,**H**) Annexin V-PI staining of 786-O cells (**G**) and Caki-1 (**H**) cells transfected with PYCR1 siRNAs or none by flow cytometry.

Complementing these observations, colony formation assays and wound healing assays demonstrated dose-dependent reductions in proliferative capacity across PYCR1-deficient ccRCC models (Figure 9A–D). Transwell invasion paradigms further revealed compromised metastatic potential through PYCR1 knockdown (Figure 9E,F). The flow cytometric quantification of Annexin V/PI staining patterns confirmed enhanced apoptotic indices in genetically modified cells (Figure 9G,H). This integrated multi-modal analysis establishes PYCR1 as a pivotal regulator of ccRCC pathophysiology, orchestrating tumor cell survival, mitotic progression, and metastatic dissemination through molecular mechanisms warranting further exploration. The collective dataset positions PYCR1 as a promising molecular vulnerability for targeted therapeutic intervention in renal malignancies.

## 3. Discussion

In previous studies focused on gene signatures for ccRCC, the clinical significance of survival-related, immune evasion-related, and metabolism-related prognostic signatures has been explored and elucidated in ccRCC patients [9,22,23]. However, these findings lacked insights on the potential crosstalk between immune evasion and metabolic reprogramming for prognosis prediction and patient stratification. Given that tumors can manipulate their metabolic pathways to promote immune escape, our study aimed to conduct a comprehensive analysis integrating immune and metabolic reprogramming-related genes [24,25]. Our findings have identified an IMRGs prognostic signature and provided new insights into the TME, drug susceptibility, and potential regulatory mechanisms in ccRCC.

Herein, we investigated the changes in the metabolic and immune landscapes in ccRCC and focused on identifying novel biomarkers for ccRCC prognosis prediction. Utilizing public datasets, we established a robust prognostic model for ccRCC based on eight IMRGs (*UCN*, *HAMP*, *SEMA3A*, *AMH*, *PYCR1*, *PLXNB3*, *CLDN4*, and *TEK*). Our results demonstrated that the IMRGs signature was significantly associated with survival probability in ccRCC patients. Notably, the high predictive accuracy of our IMRGs signature, as indicated by the AUC value of 0.776, suggesting that the IMRGs signature could serve as a reliable prognostic indicator for ccRCC patients. Importantly, we found the potential carcinogenic role of PYCR1 in the progression of ccRCC.

Beyond prognostic prediction, our study also provides insights into the TME in ccRCC. We observed distinct differences in immune cell infiltration between low- and high-risk groups stratified by our IMRGs signature. Specifically, the high-risk patient group exhibited a more active immune response, characterized by significantly lower infiltration levels of myeloid dendritic cells but higher infiltration levels of CD8 T cells. Furthermore, we identified significant associations between the IMRGs, the expression levels of immune checkpoint molecules, and TMB in ccRCC. The high-risk patient group exhibited a significantly higher TMB and higher expression levels of CTLA4 and PDCD1, along with an increased TIDE score and CTLA4-PD1-based IPS, but lower expression of CD274. The immune cell composition and activation status, TMB, and the expression levels of immune checkpoint molecules were closely correlated with the efficacy of immunotherapy, which may partly explain the significant difference in risk score between the responders and non-responders to immunotherapy [26,27,28]. Considering the urgent need for effective biomarkers for predicting the response to ICIs, the IMRGs signature may serve as a valuable tool in predicting treatment sensitivity. Further investigations are needed to elucidate the functional roles of specific immune cell subsets and to optimize the current model for predicting immunotherapeutic response in ccRCC.

In our study, GSEA analysis indicated the significant differences in the TME, immune response and metabolic characteristics between high-risk and low-risk groups. The high-risk group exhibited enrichment in pathways such as cytokine–cytokine receptor interaction, NOD-like receptor signaling pathway, and primary immunodeficiency. Cytokine–receptor interactions within the TME participate in modulating immune responses and facilitating the communication between tumor cells and the immune microenvironment, which often result in muted immune responses that facilitate tumor progression [29]. Additionally, previous studies have revealed that NOD1 initiates the secretion of inflammatory cytokines and chemokines, which contribute to tumor development and progression [30]. The enrichment of the primary immunodeficiency pathway suggests a diminished immune surveillance function, which makes it easier for tumor cells to evade recognition and attack by the immune system. Above all, the high-risk group displayed stronger immune cell signaling and inflammatory responses, but may be associated with immune escape mechanisms. The enrichment of these pathways in the high-risk group underlines their potential as therapeutic targets and highlights the importance of further explaining their roles in ccRCC treatments.

The eight IMRGs with prognostic significance identified in this study were *AMH*, *CLDN4*, *HAMP*, *SEMA3A*, *PLXNB3*, *UCN*, *PYCR1*, and *TEK*. The expression levels of these genes were found to be upregulated in the renal cancer cell lines. Among these genes, *AMH*, *CLDN4*, *HAMP*, *PLXNB3*, *UCN*, and *TEK* have been incorporated into several immune-related gene signatures for predicting clinical outcomes and survival in ccRCC, yet their specific roles in ccRCC remain to be fully elucidated [31,32,33,34,35]. *SEMA3A* can cause immune suppression by affecting tumor-specific CD8^+^ T cell filamentous actin, which results in inhibition of immune synapse formation and motility [36]. *PYCR1*, on the other hand, has been included in a metabolism-related gene signature and has been shown to regulate glutamine metabolism, thereby constructing an immunosuppressive microenvironment that facilitates ccRCC progression [37,38]. Given its well-recognized oncogenic properties, *PYCR1* was selected for further investigation to elucidate its role in ccRCC. Previous studies have shown that *PYCR1* functions as an oncogene and promotes malignant progression in various tumors, such as lung and liver cancers [16,39]. Consistent with these findings, our findings identify *PYCR1* as a pivotal regulator of ccRCC progression, influencing tumor cell proliferation and migration. Additionally, we found that *PYCR1* knockdown could enhance apoptosis in ccRCC cells, which is in line with previous reports [40,41]. *PYCR1* has also been reported as a target gene for 5-fluorouracil-induced ferroptosis and apoptosis in colorectal cancer, and its overexpression can confer resistance to 5-fluorouracil-induced cytotoxic effects in colorectal cancer cells [40]. These findings suggest that *PYCR1* may serve as a potential target for enhancing the efficacy of tumor chemotherapy, especially for chemotherapeutic drugs that induce apoptosis as their main mechanism. In addition, while high-risk patients exhibit increased CD8^+^ T-cell infiltration in our study, these cells likely undergo functional exhaustion, as evidenced by elevated PD-1/CTLA-4 expression and enrichment of exhaustion-related pathways. Furthermore, the high-risk group showed higher infiltration of immunosuppressive cells and elevated immunosuppressive signaling, creating an immune-hostile microenvironment that neutralizes CD8^+^ T-cell effector functions. This aligns with prior studies linking CD8+ T-cell exhaustion to poor prognosis despite high infiltration [42,43].

Current prognostic models or scores have been explored extensively in many studies. For example, Büttner et al. developed a gene expression-based prognostic score (S3-score) for ccRCC, using the cell type-specific expression of 97 genes within the human nephron. By integrating the TCGA database, a 15-gene simplified version of the score (S315) applicable to the qRT-PCR platform was developed. Its efficacy was verified in multiple independent cohorts containing primary/metastatic lesions and patients treated with sunitinib. The results showed that the S315 score could independently predict the cancer-specific survival of metastatic/non-metastatic patients (HR = 5.0, *p* = 5.1 × 10⁻^5^), and was consistent with the prognosis prediction of metastatic foci, significantly superior to the traditional clinical score SSIGN (*p* = 1.6 × 10⁻^3^), providing a new molecular stratification tool for the full-process management of ccRCC [44]. Serie et al. analyzed the primary and metastatic tissues of 111 patients with ccRCC, and for the first time revealed that approximately 22% of metastatic ccRCC had intratumoral molecular heterogeneity (ccA/ccB subtypes), and the primary and metastatic subtypes of 43% of patients were inconsistent, suggesting that the primary tumor cannot replace the metastatic lesion for molecular typing. The study further found that the subtype of metastatic foci was significantly correlated with tumor necrosis, grade and metastatic location, providing a basis for precise treatment driven by independent molecular characteristics of metastatic foci. However, it should be noted that this conclusion is limited to only including the patient group that underwent both nephrectomy and resection of metastatic foci simultaneously [45]. Rini et al. developed and validated a 16-gene recurrence score (11 prognostic + 5 reference genes) using RT-PCR in 942 stage I-III ccRCC patients, demonstrating its independent prognostic value for postoperative recurrence risk (HR 3.37 per 25-unit score increase, *p* < 0.0001) and ability to reclassify high-risk stage I/low-risk stage II-III subgroups beyond clinical parameters. Validated in 626 patients, the score showed minimal intratumoral heterogeneity and improved personalized risk stratification, offering molecular refinement to traditional prognostic tools like tumor stage and Leibovich score [46]. Our study specifically aimed to explore the underexplored intersection of immune and metabolic pathways in ccRCC prognostication, a dimension not systematically addressed in prior models. While existing scores excel in integrating clinicopathological variables and pan-genomic markers, our IMRGs provides novel biological insights into tumor microenvironment dynamics and therapeutic vulnerabilities, particularly for immunotherapy response prediction.

Several limitations should be acknowledged in our study. Regarding the potential limitation of the TCGA_KIRC cohort, due to misclassifications, each of the TCGA_RCC cohorts also comprises other RCC subtypes. Especially, about 10% of samples in the KIRC cohort are indeed of papillary, chromophobe, or mixed type [47,48]. Future studies incorporating centralized pathology review or single-subtype cohorts will help resolve this issue. The retrospective design and limited cohort sizes inherent in this investigation may engender selection biases and constrain statistical power. To address these methodological limitations, prospective validation through large-scale multicenter trials and longitudinal surveillance initiatives will be imperative for establishing generalizable validation frameworks. Current prognostic model as continuous variables used the dichotomization of the risk score by the median may have some drawbacks and should therefore generally be evaluated in further studies. In addition, our machine learning framework employed a 70:30 stratified split to balance clinical interpretability with computational feasibility in a relatively small size dataset. Nested cross-validation or bootstrapping in larger cohorts may further improve generalizability estimates. The current stratification paradigm employing IMRG-derived cut-off values necessitates refinement through advanced machine learning algorithms and dynamic risk modeling approaches to optimize clinical stratification accuracy. Mechanistically, the pathobiological interplay between identified immune–metabolic regulators and canonical oncogenic signaling cascades remains to be fully deciphered through integrated multi-omics profiling and CRISPR-based functional genomics platforms. Future studies will delineate PYCR1’s downstream targets and validate its in vivo tumor-promoting effects.

In summary, our work systematically developed and validated a composite prognostic index integrating immune–metabolic molecular signatures with clinicopathological parameters for ccRCC risk stratification. The biological relevance and therapeutic implications of this stratification framework were substantiated through multidimensional bioinformatics interrogation and experimental validation. Notwithstanding these contributions, the molecular circuitry connecting IMRG dysregulation with tumor microenvironment remodeling merits comprehensive exploration using spatial transcriptomics and single-cell sequencing technologies. These foundational insights establish a conceptual framework for advancing precision oncology in renal malignancies, potentially informing the development of molecularly guided therapeutic paradigms and biomarker-driven clinical trial designs to improve ccRCC management.

## 4. Materials and Methods

### 4.1. Acquisition and Processing of Datasets

The genomic landscape of kidney renal clear cell carcinoma (KIRC) was interrogated through comprehensive data retrieval from The Cancer Genome Atlas (TCGA) portal, encompassing RNA sequencing profiles, clinical annotations, survival records, and microsatellite instability status from the TCGA Xena repository. Patients were stratified by age, gender, TNM stage, and tumor grade to ensure proportional representation between training (70%) and validation (30%) cohorts, ensuring the proportional representation of critical clinical variables. A differential gene expression analysis between malignant and adjacent normal tissues was performed using the ‘limma’ package (version 3.63.13) with rigorous multiple testing correction (|logFC| > 1 and false discovery rate-adjusted *p* < 0.05).

### 4.2. Collection of Metabolism and Immune-Related Gene Sets

Curated gene sets for immunophenotyping and metabolic profiling were systematically compiled through the integration of authoritative resources: 2483 immune-related genes derived from the ImmPort immunological database (https://www.immport.org, accessed on 5 May 2024) and 948 metabolism-associated genes extracted from “c2.cp.kegg.v7.4. symbols” from MSigDB (v7.4).

### 4.3. Construction and Validation of IMRGs Signature

The “ns non-negative matrix factorization (nsNMF)” algorithm was selected with 100 iterations performed and the number of clusters K was set in the range of 2 to 10 to stratify ccRCC patients into two distinct molecular subtypes. Then, the univariate and least absolute shrinkage and selection operator (LASSO) Cox regression analyses using the R package ‘glmnet’ (version 4.1-8) were employed to identify a subset of prognostic genes associated with ccRCC from a panel of the DEGs. Combining the data files of gene expression and patient survival, a list of prognostic genes with correlation coefficients were obtained according to the best lambda. Risk scores were computed by summing the expression of each selected gene with its corresponding coefficient. Patients were categorized into low- and high-risk groups based on the median risk score. The prognostic performance of the signatures was assessed using Kaplan–Meier analysis, time-dependent receiver operating characteristic (ROC) analysis, and the C-index. The nomograms were constructed to evaluate the survival probability for patients at 1-, 3- or 5-year intervals by using the R package ‘rms’ (version 8.0-0). The time-dependent ROC curves were performed using the R package ‘timeROC’ (version 0.4), to evaluate the sensitivity and specificity of the signature for predicting the prognosis of ccRCC patients.

### 4.4. Gene Set Enrichment Analysis (GSEA)

The GSEA was performed based on the KEGG gene sets by the GSEA software (v4.0.1). The gene sets in the results were considered significant if the conditions of |NES| >  1, NOM *p* value  <  0.05, and FDR q value  <  0.25 were met.

### 4.5. Evaluation of the Tumor Microenvironment and Response to Immunotherapy

The microenvironment cell populations-counter (MCP-counter) method was used for calculating the immune cell populations and immune scores in the different molecule subtypes [49]. The CIBERSORT algorithm was utilized to investigate immune infiltration and function between low- and high-risk groups based on the prognostic signature [50]. Additionally, the expression of immune checkpoints was evaluated and compared between groups utilizing the Kruskal–Wallis rank sum test. The tumor immune dysfunction and exclusion (TIDE) algorithm (http://tide.dfci.harvard.edu/login/, accessed on 5 May 2024) [51,52] was used to estimate a tide score and the predicted response to immune checkpoint blockade. Immunophenoscore (IPS) was also calculated using the four main factors, including MHC molecules, immunomodulators, effector cells, and suppressor cells, in the TCIA database [53] (https://tcia.at/home, accessed on 5 May 2024), which was used to predict the therapeutic responses to the four major immune checkpoints (including PD-1 and its two ligands, PD-L1/PD-L2 as well as CTLA-4). Moreover, we analyzed the correlation of IMRGs signature with microsatellite instability (MSI) and tumor mutational burden (TMB), indicators used to reflect the efficacy of immunotherapy.

### 4.6. Comparisons of Drug Sensitivity

Half-maximal inhibitory concentration (IC50) values for the most used chemotherapeutic medicines were calculated using the ‘pRRophetic’ R package (version 0.5). The drug sensitivity of one risk group was compared to the other, and any statistically significant differences were tested using the Wilcox test [54,55]. Furthermore, we collected the IC50 of 265 small molecules in 860 cell lines and its corresponding mRNA gene expression from Genomics of Drug Sensitivity in Cancer (GDSC) [20]. We also collected the IC50 of 481 small molecules in 1001 cell lines and its corresponding mRNA gene expression from the Cancer Therapeutics Response Portal (CTRP) [56]. The mRNA expression data and drug sensitivity data were merged. A Pearson correlation analysis was performed to obtain the correlation between gene mRNA expression and drug IC50. The *p*-value was adjusted by FDR.

### 4.7. Cell Lines and Culture

Human renal cell line, namely HK-2 (IM-H060), and human clear cell renal cell carcinoma cell lines, namely Caki-1 (IM-H364) and 786-O (IM-H055), were purchased from Immocell Biotechnology (Xiamen, China), and maintained in DMEM (Gibco, Waltham, MA, USA) supplemented with 10% fetal bovine serum (Ausbian, Adelaide, Australia) at 37 °C with 5% CO_2_, respectively.

### 4.8. RNA Isolation and Quantitative Reverse Transcription Polymerase Chain Reaction (qRT-PCR)

Total RNAs were isolated from Caki-1, 786-O, and HK-2 cells by TRIzol (Sangon, Shanghai, China) according to the protocol. qRT-PCR was performed using an SYBR Green PCR Kit (Takara, Shiga, Japan) on an ABI 7500 System. GAPDH was employed as a control for normalization when mRNA expression was detected. Each experiment was repeated at least three times, and the data were analyzed using the 2 ^(−△△Ct)^ method. Transcriptional copy number was measured by using standard curve method and the exact copy numbers of risk model genes (*UCN*, *HAMP*, *SEMA3A*, *AMH*, *PYCR1*, *PLXNB3*, *CLDN4*, and *TEK*) transcript were calculated by relating the Ct value to standard curve. Primers are listed in Table S1 and were synthesized by Tsingke Biotechnology (Beijing, China).

### 4.9. Construction and Infection of Lentivirus Plasmid

Through the NCBI website (https://ncbi.nlm.nih.gov/, accessed on 12 July 2024), to obtain the purpose gene PYCR1 mRNA Sequence (NCBI Reference Sequence: NM_001282281.2), select appropriate knockdown sequences according to the principles of siRNA sequence design, and design three shRNA interference sequences (Table S2). ShPYCR1 primer and lentivirus knockdown plasmid pLKO.1-puro-shRNA plasmid and lentivirus packaging assistant plasmid were obtained from Tianwei Biotechnology (Hangzhou, China). Negative control (NC) shRNA (sh-NC) served as the control of shPYCR1. First, the plasmid was double-cut and then connected to pLKO.1-puro-shRNA lentiviral vector plasmid by AgeI and EcoRI cleavage sites. Sequence-validated constructs were transformed into Stbl3 competent cells for plasmid amplification and endotoxin-free purification.

After suspension, the cultured 293T cells were placed in a 15 mL centrifuge tube and counted after diluting the number of cells by an appropriate multiple. According to PEI operating guidelines, pLKO.1-puro-shRNA: pSpax2: pMD2.G = 5:5:2 was transfected. Viral supernatants harvested at 48 h post-transfection underwent 0.45 μm filtration and ultracentrifugation (20,000 g, 2 h) for concentration. Caki-1 and 786-O renal carcinoma cells (1 × 10^5^ cells/mL) were transduced with viral particles in medium containing 8 μg/mL polybrene. Stable transfectants were selected through 48 h puromycin treatment (1.5 μg/mL) initiated 120 h post-infection, followed by knockdown efficiency validation.

### 4.10. Protein Extraction and Western Blot

Cellular proteins were isolated using RIPA lysis buffer (Beyotime, Shanghai, China) and quantified through BCA assay. Equal protein aliquots (20 μg) underwent electrophoretic separation via 10% SDS-PAGE before electrotransfer to PVDF membranes (Millipore, Burlington, MA, USA). Membranes were blocked with 5% BSA for 1 h at room temperature followed by 4 °C overnight incubation with primary antibodies against PYCR1 (1:1000, #Cat: 13108-1-AP, Proteintech, San Diego, CA, USA) and β-actin (1:5000, #Cat: 66009-1-Ig, Proteintech). After three TBST washes, the membranes were probed with HRP-conjugated secondary antibodies (1:5000) for 1 h at room temperature. Protein bands were visualized using the ImageQuant LAS 4000 mini chemiluminescent detection system (General Electric Company, Boston, MA, USA), with β-actin serving as the loading control.

### 4.11. Cell Growth and Colony Formation Assays

Cell viability was assessed using the CCK-8 assay. In the CCK8 experiment, 96-well plates were laid with 3000 plates per hole, and 3 double holes and a blank control were set. At 0 h from the next day, 10 μL CCK8 reagent (GLPBIO, Montclair, CA, USA) was added to each well at 0 h, 24 h, 48 h, and 72 h, respectively, and the photometric value was measured at 450 nm after incubation for 2 h. For cell colony formation assays, 24 h after transfection, 800 Caki-1 and 786-O cells were incubated in 6-well plates at 37 °C, 5% CO_2_. Twelve days later, the cells were stained with crystal violet (0.2%) for 30 min and the colony numbers were counted.

### 4.12. Wound Healing Assay

Cells were seeded in a 6-well plate and allowed to reach approximately 90% confluence. Using a sterile 10 μL pipette tip, a wound was created on the cell monolayer. The cells were then cultured in basal medium, and photographs were taken at 0 h, 24 h, and 48 h under a microscope to record the wound width.

### 4.13. Transwell Assays

Several matrix adhesives were diluted 10-fold and aliquoted (70 μL/well) into the upper chambers of Transwell inserts, followed by incubation at 37 °C with 5% CO_2_ for 5 h to allow polymerization. Cells were harvested, centrifuged, and resuspended in serum-free DMEM. Subsequently, 100 μL of cell suspension (1 × 10^5^ cells) was seeded into the upper chamber of a 24-well Transwell system, while 600 μL of DMEM supplemented with 10% FBS was added to the lower compartment. After 24 h incubation under standard culture conditions (37 °C, 5% CO_2_), the Transwell membranes were gently rinsed twice with PBS and fixed in 4% paraformaldehyde for 5 min. Following PBS washes, non-migrated cells on the upper membrane surface were removed by cotton swab abrasion. The migrated cells were air-dried, stained with 0.1% crystal violet for 15 min, and thoroughly rinsed with PBS. Membranes were then visualized and imaged using bright-field microscopy.

### 4.14. Cell Apoptosis by Flow Cytometry

Caki-1 and 786-O cells were collected 24 h post-transfection through trypsinization, washed with ice-cold PBS, and fixed in 70% ethanol for 24 h at 4 °C. For cell cycle analysis, ethanol-fixed cells were stained with propidium iodide (PI) for 30 min in darkness and analyzed using a FACScan flow cytometer (BD Biosciences, San Diego, CA, USA). Data were processed with ModFit LT software (version 5.0) (Verity Software House, Lexington, MA, USA). Apoptosis was quantified using the Annexin V-FITC/PI Apoptosis Detection Kit (BD Biosciences, USA), where dual-stained cells were subjected to flow cytometric analysis. Apoptosis data were interpreted using FlowJo software (version 10.8.1) (Tree Star, Ashland, OR, USA).

### 4.15. Statistical Analysis

Statistical analysis was performed using R language (version 4.1.0) and GraphPad Prism 8.0. All the experiments were repeated at least three times, and the collected data are presented as the mean ± SD. The data between the two groups were compared using the *t*-test. The Wilcoxon signed-rank test was employed to compare the expression of PYCR1 mRNA between tumor tissues and normal tissues. A one-way analysis of variance (ANOVA) was used for comparisons among multiple groups. Spearman correlation analysis was applied to explore the correlation between continuous variables. Single-factor Cox proportional hazards regression model or log-rank test was used to assess the correlation between gene expression and the overall survival rate of patients. A significance level of *p* < 0.05 was considered statistically significant.

## Figures and Tables

**Table 1:** Gene information of identified risk model.

Gene Name	Full Name	Functions	Immunity or Metabolism Gene
*UCN*	Urocortin	Activation of cAMP-dependent PKA and peptide hormone metabolism	Immunity
*HAMP*	Hepcidin antimicrobial peptide	Hfe effect on hepcidin production and TAR syndrome	Immunity
*SEMA3A*	Semaphorin 3A	Apoptotic pathways in synovial fibroblasts and GPCR pathway	Immunity
*AMH*	Anti-mullerian hormone	Mammalian disorder of sexual development and signaling by TGFB family members	Immunity
*PYCR1*	Pyrroline-5-Carboxylate Reductase 1	Glutamate and glutamine metabolism and superpathway of L-citrulline metabolism	Metabolism
*PLXNB3*	Plexin B3	Nervous system development and semaphorin interactions	Immunity
*CLDN4*	Claudin 4	Blood–brain barrier and immune cell transmigration: VCAM-1/CD106 signaling and cell junction organization	Immunity
*TEK*	TEK Receptor Tyrosine Kinase	GPCR pathway and ERK signaling	Immunity

ERK: extracellular signal-regulated kinase; GPCR: G protein-coupled receptor; Hfe: human hemochromatosis protein; PKA: protein kinase A; TAR: thrombocytopenia absent radius; TGFB: transforming growth factor beta; and VCAM-1/CD106: vascular cell adhesion molecule 1.

## Data Availability

The datasets analyzed during this study are available in the TCGA database (https://portal.gdc.cancer.gov, accessed on 5 May 2024) (TCGA KIRC). The datasets utilized or analyzed in the current study are also available from the corresponding authors at reasonable request.

## Supplementary Materials

The following supporting information can be downloaded at: https://www.mdpi.com/article/10.3390/ijms26104953/s1.
