# Supplementary material for: Integrative Analysis of Immune- and Metabolism-Related Genes Identifies Robust Prognostic Signature and PYCR1 as a Carcinogenic Regulator in Clear Cell Renal Cell Carcinoma
Source: Int J Mol Sci. 2025 May 21;26(10):4953. doi: 10.3390/ijms26104953 (PMC12112471; doi:10.3390/ijms26104953)
Supplement: Supplementary file 1 [file ijms-26-04953-s001.zip › ijms-3567452-supplementary.pdf]

Supplementary materials

Development and validation of an immune- and metabolism-related gene signature for prognosis and immunotherapy response in clear cell renal cell carcinoma

Guo Zhao [1,#], Jiatong Ding [1,#], Jiaxiu Ma [2], Yale Jiang [1], Yuning Wang [1], Shuhang Wang [1,*] and Ning Li [1,*]

[1] Clinical Trial Center, National Cancer Center/National Clinical Research Center for Cancer/Cancer Hospital, Chinese Academy of Medical Sciences and Peking Union Medical College, Beijing 100021, China.

[2] State Key Laboratory of Experimental Hematology, National Clinical Research Center for Blood Diseases, Haihe Laboratory of Cell Ecosystem, Institute of Hematology and Blood Diseases Hospital, Chinese Academy of Medical Sciences and Peking Union Medical College, Tianjin, China

[#]These authors contributed equally to this work.

*Corresponding authors:

S. Wang (wangshuhang@cicams.ac.cn)

N. Li (lining@cicams.ac.cn)

Supplementary Table S1. Design of primers of PYCR1

Primer Name	Primer sequence (5' → 3')
sh*PYCR1*-1U	CCGGCACAGTTTCTGCTCTCAGGAACTCGAGTTCCTGAGAGCAGAAACTGTGTTTTTG
sh*PYCR1*-1L	CAAAAACACAGTTTCTGCTCTCAGGAACTCGAGTTCCTGAGAGCAGAAACTGTGCCGG
sh*PYCR1*-2U	CCGGGAGGGTCTTCACCCACTCCTACTCGAGTAGGAGTGGGTGAAGACCCTCTTTTTG
sh*PYCR1*-2L	CAAAAAGAGGGTCTTCACCCACTCCTACTCGAGTAGGAGTGGGTGAAGACCCTCCCGG
sh*PYCR1*-3U	CCGGTGAGAAGAAGCTGTCAGCGTTCTCGAGAACGCTGACAGCTTCTTCTCATTTTTG
sh*PYCR1*-3L	CAAAAATGAGAAGAAGCTGTCAGCGTTCTCGAGAACGCTGACAGCTTCTTCTCACCGG

Supplementary Table S2. Design of fluorescent quantitative primers

Primer Name	Primer sequence (5' → 3')
qUCN-F	TGTGGCTGTCATTGCTTCTAC
qUCN-R	GTCTGTACGGTCCAAGATTGAG
qHAMP-F	CGCTTGCCTCCTGCTCCT
qHAMP-R	CTCGCCTCCTTCGCCTCT
qSEMA3A-F	GTGCCAAGGCTGAAATTATCCT
qSEMA3A-R	CCCACTTGCATTCATCTCTTCT
qAMH-F	AGCTGTGGGCACCAGTGG
qAMH-R	GCTCTTGTGGGCTGCCTG
qPYCR1-F	ACACCCCACAACAAGGAGAC
qPYCR1-R	CTGGAGTGTTGGTCATGCAG
qPLXNB3-F	CGCTTCTCCGCACCTAATACC
qPLXNB3-R	CAGGGCTGTCGATTACAGGG
qCLDN4-F	GAGGGCCTATGGATGAACTG
qCLDN4-R	AGCAGCGAGTCGTACACCTT
qTEK-F	TTAGCCAGCTTAGTTCTCTGTGG
qTEK-R	AGCATCAGATACAAGAGGTAGGG
qhActb-F	ACGTGGACATCCGCAAAGAC
qhActb-R	TCTTCATTGTGCTGGGTGCC

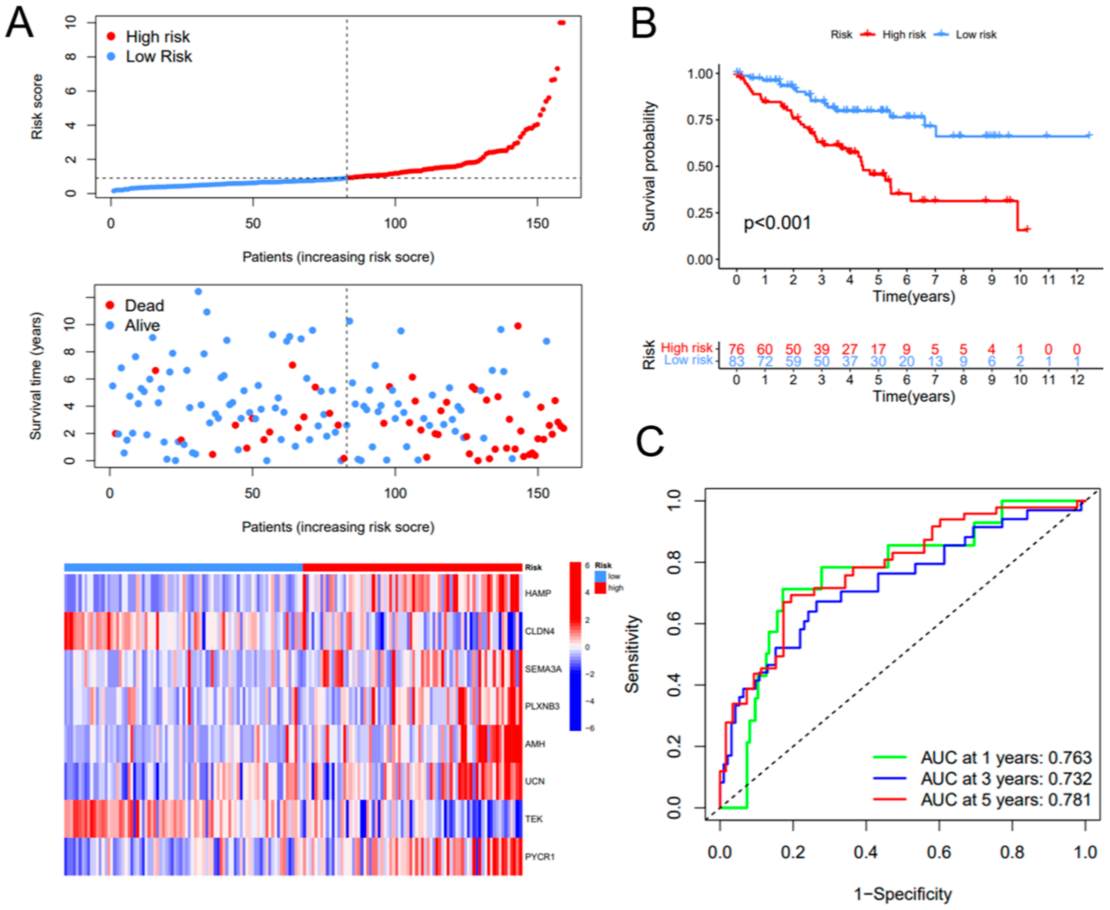

Supplementary Figure S1. Validation of the IMRGs prognostic signature in TCGA KIRC testing cohorts. (**A**) Distribution of risk scores and survival status in TCGA testing cohorts. Heatmap of the gene-expression profiles of the IMRGs prognostic signature; (**B**) Kaplan–Meier curve analyses for high-risk group and low-risk group; (**C**) ROC curves of the IMRGs prognostic signature at 1-, 3-, and 5-year.

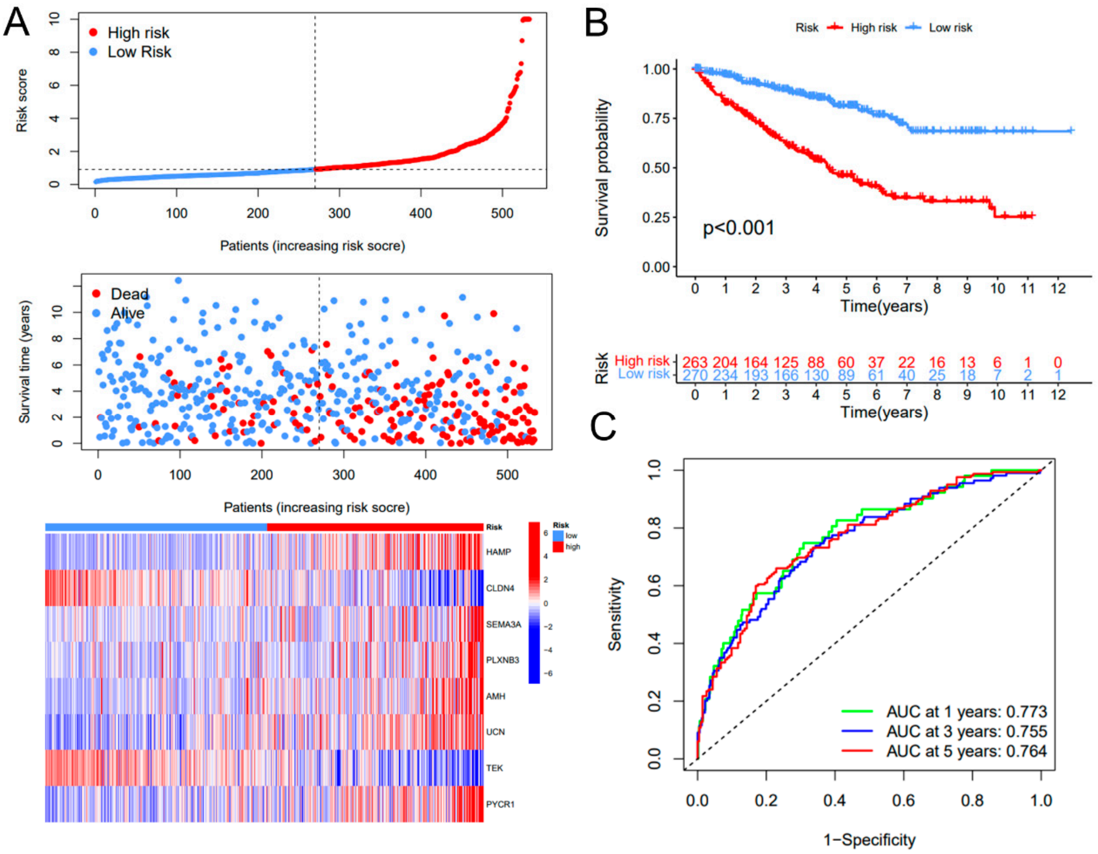

Supplementary Figure S2. Validation of the IMRGs prognostic signature in entire TCGA KIRC cohorts. (**A**) Distribution of risk scores and survival status in entire TCGA KIRC cohorts. Heatmap of the gene-expression profiles of the IMRGs prognostic signature; (**B**) Kaplan–Meier curve analyses for high-risk group and low-risk group; (**C**) ROC curves of the IMRGs prognostic signature at 1-, 3-, and 5-year.

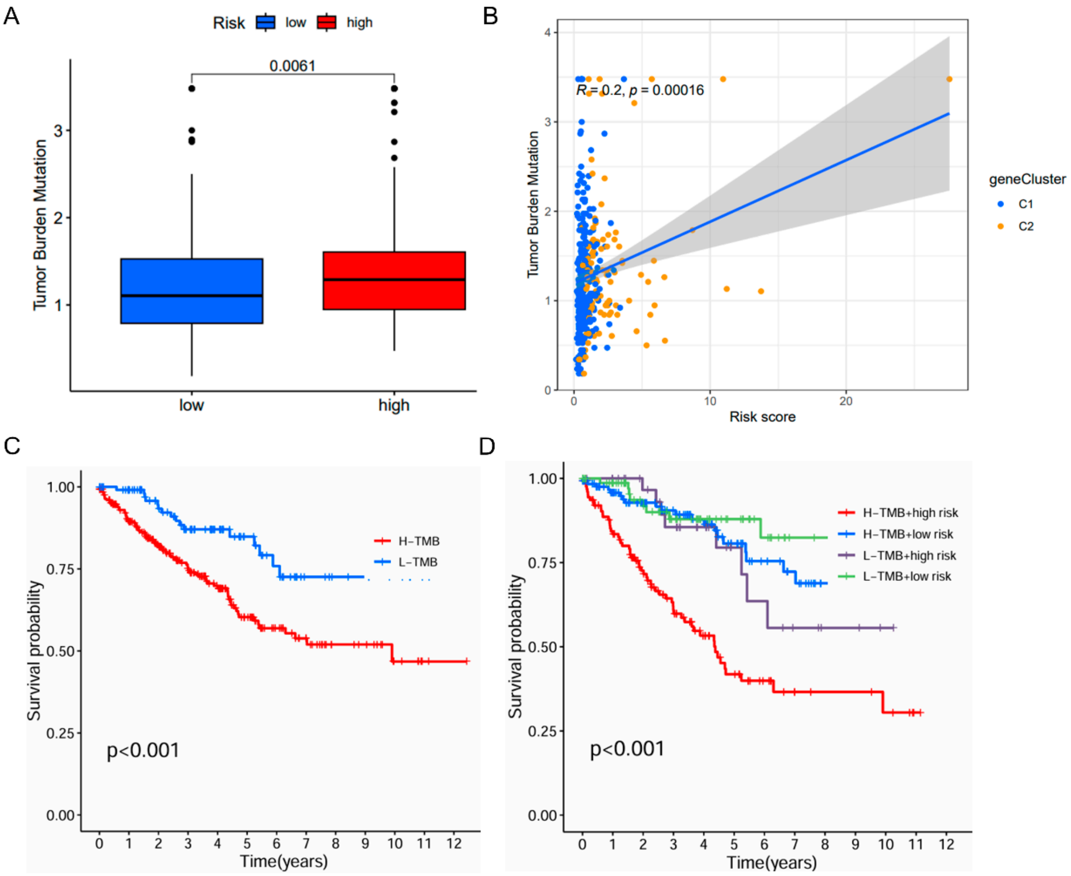

Supplementary Figure S3. Correlation of the IMRGs prognostic signature and tumor mutational burden (TMB). (**A**) Comparison of risk scores and TMB scores between high- and low- risk groups; (**B**) Relationship of risk scores and TMB scores between molecule subtypes; (**C**) Kaplan–Meier curve analyses for high-TMB group and low- TMB group; (**D**) Kaplan–Meier curve analyses of the risk scores and TMB scores.

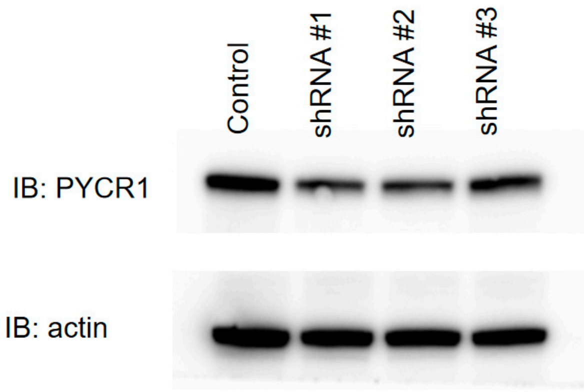

Supplementary Figure S4. Western blot of shRNA screening of shPYCR1 cells
